# MIS-11 duration key to disappearance of the Greenland ice sheet

**DOI:** 10.1038/ncomms16008

**Published:** 2017-07-06

**Authors:** Alexander Robinson, Jorge Alvarez-Solas, Reinhard Calov, Andrey Ganopolski, Marisa Montoya

**Affiliations:** 1 Universidad Complutense de Madrid, 28040 Madrid, Spain; 2 Instituto de Geociencias, CSIC-UCM, 28040 Madrid, Spain; 3 Potsdam Institute for Climate Impact Research, 14473 Potsdam, Germany

## Abstract

Palaeo data suggest that Greenland must have been largely ice free during Marine Isotope Stage 11 (MIS-11). However, regional summer insolation anomalies were modest during this time compared to MIS-5e, when the Greenland ice sheet likely lost less volume. Thus it remains unclear how such conditions led to an almost complete disappearance of the ice sheet. Here we use transient climate–ice sheet simulations to simultaneously constrain estimates of regional temperature anomalies and Greenland’s contribution to the MIS-11 sea-level highstand. We find that Greenland contributed 6.1 m (3.9–7.0 m, 95% credible interval) to sea level, ∼7 kyr after the peak in regional summer temperature anomalies of 2.8 °C (2.1–3.4 °C). The moderate warming produced a mean rate of mass loss in sea-level equivalent of only around 0.4 m per kyr, which means the long duration of MIS-11 interglacial conditions around Greenland was a necessary condition for the ice sheet to disappear almost completely.

## Abstract (web summary)

The most pronounced deglaciation of Greenland of the last several hundred thousand years occurred during Marine Isotope Stage 11. Here, the authors show that it was the exceptional duration of the interglacial period that allowed Greenland to melt even though temperatures were only moderately warmer than today.

The globally averaged MIS-11 sea level is estimated to have reached between 6–13 m above that of today^1^. With only a small contribution from thermal expansion of the ocean, this implies that significant parts of either or both the Greenland ice sheet (GrIS) and the West Antarctic ice sheet disappeared during this time. While direct evidence of ice-sheet volume and area changes during past interglacial periods is generally not available, several palaeo records point to a significant reduction of the GrIS during MIS-11.

Ancient DNA has been identified in the silt beneath the DYE-3 ice core pertaining to several boreal forest species including Alder, Spruce and Pine, as well as that of some insects^2^. The existence of such material, most likely local in origin, indicates that most or all of southern Greenland, and particularly the DYE-3 location, was ice free for a period of time. Approximate dating of this material suggests that it predates the last interglacial period (ca. 130–115 kyr BP), which makes MIS-11 a likely candidate. Recent dating of air trapped near the base of the ice core also suggests that the oldest ice at DYE-3 may have appeared at the end of MIS-11, though this result is highly uncertain^3^.

This finding is further supported by oceanic sediment records. Pollen obtained from cores off the southern coast of Greenland corroborate the existence of a pronounced increase in abundance of several species of boreal vegetation^4^. The high concentrations of pollen and the proximity of the core make southern Greenland the most likely source. In addition, analysis of a core on the Eirik Drift shows a cessation of proglacial sediment deposition during MIS-11, consistent with retreat of the ice sheet from most bedrock terrane boundaries of southern Greenland^5^, that is, south of 69 °N.

Meanwhile, air trapped in the basal layers of the GRIP ice core (drilled at the present-day summit of the ice sheet) has been estimated to date to around 1 Myr BP^3^. Furthermore, analysis of cosmogenic elements in the soil beneath the ice sheet shows that the glacial landscape there has been preserved over the last few million years^6^. These data indicate that it is unlikely that this location became ice free for any extended period of time since the glacial inception of Greenland.

The above palaeo information provides complementary constraints on the Greenland climate and ice-sheet extent during this time. The DNA and pollen records provide information about local climatic conditions over southern Greenland, while the sediment records, ice core dating and the summit landscape dating give lower and upper bounds, respectively, to changes in ice area in key locations.

Indeed, Reyes *et al*.^5^ recently proposed that a large sea-level contribution from Greenland of 4.5–6 m during this time would be consistent with the above evidence. Their conclusion was obtained by comparing the constraints with published simulations of GrIS retreat in different scenarios (MIS-5e, global warming). However, no regional ice sheet model simulations have yet been performed for Greenland under MIS-11 climatic conditions, precluding a rigorous comparison with the data until now.

Most importantly, what also remains unclear is the key factor responsible for the decline of the ice sheet. Insolation was not anomalously high compared to today throughout most of MIS-11, and temperatures in the region were likely not as high as during MIS-5e (ref. 4)—the most recent period when the GrIS lost significant volume. However, temperatures most likely did stay warmer than present around Greenland for much longer than during MIS-5e (refs 4, 5, 7). As suggested by Reyes *et al*.^5^, this may mean that the GrIS crossed a stability threshold in temperature that allowed the ice sheet to decline over several thousand years. In general, the rate of ice-sheet decline is proportional to the magnitude of the temperature anomaly above such a stability threshold—estimated for Greenland to be around 1–2 °C given present-day insolation^8^.

In order to better understand this time period, we use the above palaeo information to constrain an ensemble of simulations of the GrIS through MIS-11. The coupled climate–ice-sheet model REMBO-SICOPOLIS^9^,^10^ is used to complete transient simulations that start at 550 kyr BP (to properly initialize the ice sheet) and run through MIS-11 until glacial conditions are reached again. Simulations are considered valid when the GRIP location always remains ice covered and the DYE-3 location becomes ice free at some point during the warm period. In addition, the simulations that exhibit more ice-free area south of 69 °N are considered to be more likely. A 300-member ensemble consisting of perturbed model parameters (two parameters) and temperature scaling (one parameter) was generated via Latin Hypercube sampling^11^. By applying the palaeo constraints described above, we checked the plausibility of each simulation and generated probabilisitic estimates of both the climatic forcing and ice-sheet evolution (see Methods for more details).

## Results

### Greenland during MIS-11

**Figure 1:**
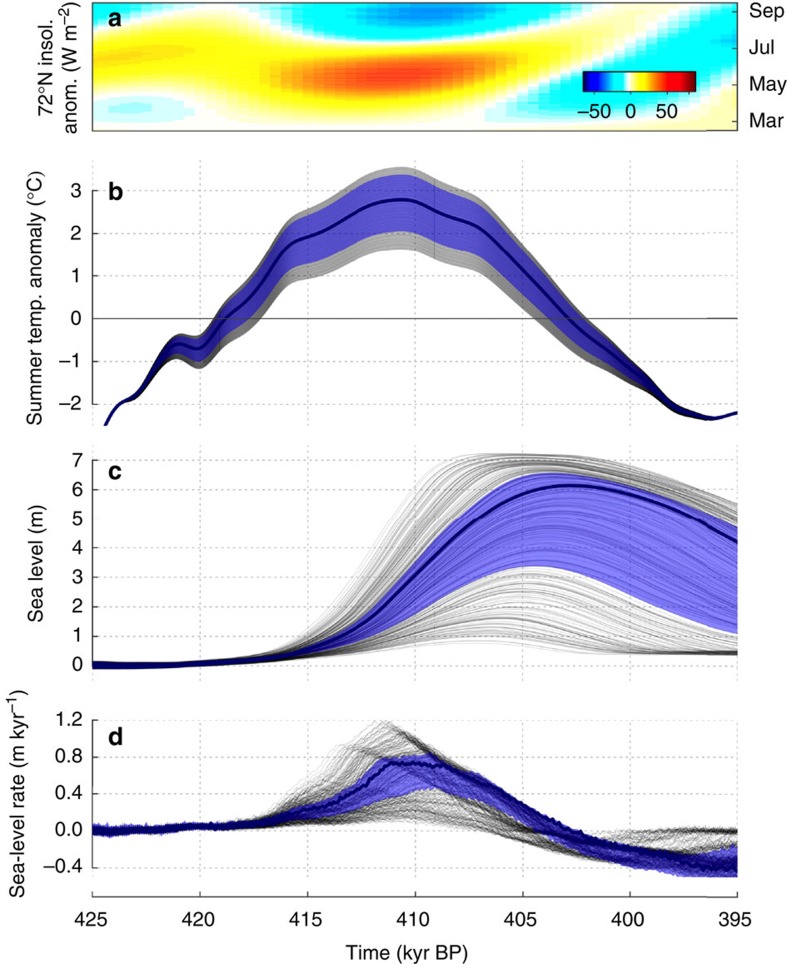
MIS-11 time series. Insolation anomaly (W m^−2^) for March-September at 72 °N (**a**) and regional summer (JJA) temperature anomaly (°C) forcing (**b**) relative to present day, simulated sea-level equivalent volume loss (m s.l.e.) of the Greenland ice sheet (**c**) and simulated rate of sea-level contribution (m per kyr) (**d**). In **b**–**d**, the grey lines show the individual simulations, while the shaded blue band and thick blue line indicate the constrained 95% credible interval and the most likely simulation, respectively.

**Figure 2:**
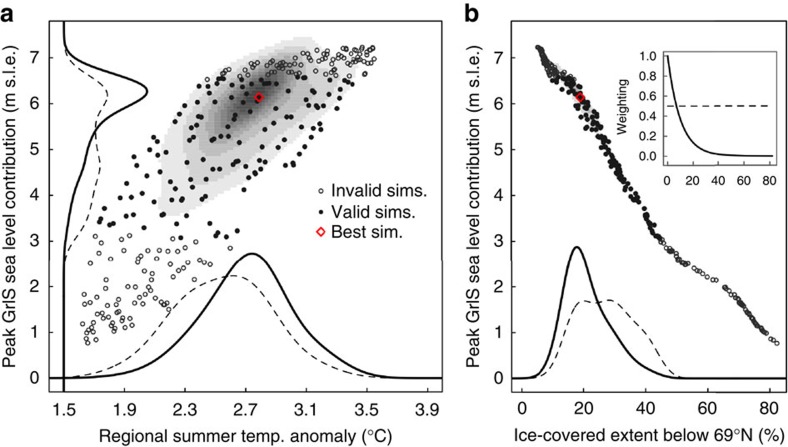
MIS-11 peak estimates. Posterior probability distributions for the maximum Greenland ice sheet contribution to sea level versus the maximum regional summer temperature anomaly (**a**) and the minimum ice cover south of 69 °N (**b**). Invalid (open circles) and valid (solid circles) simulations are shown, as well as the most likely simulation (red diamond). The shading represents the 2D probability density estimate which combines the prior ensemble with the posterior weighting as described in the text, while the lines show the 1D equivalent. The exponential weighting function (solid line in the inset) was used by default, while uniform weighting was also tested (dashed lines), giving similar locations of the probability density maxima.

The MIS-11 regional positive summer (June–July–August mean) temperature anomalies in our simulations are coincident with positive anomalies in summer insolation peaking near 411 kyr BP (Fig. 1). The applied summer temperature anomalies range from 1.6 to 3.6 °C, while the duration of these anomalies above 0 °C ranges from 12.8 to 17.5 kyr. The wide range of interglacial trajectories sampled result in a GrIS response ranging from almost no mass loss to complete deglaciation. The application of the palaeo constraints to the ice-sheet extent (DYE-3 and southern Greenland ice free, GRIP ice covered), however, limits the magnitude and duration of summer temperature anomalies to a range that reflects the fact that temperatures needed to be high enough for a long enough period of time to melt ice away from southern Greenland, but not high enough to melt the ice sheet completely (Fig. 1). From the constrained simulations, we estimate that the peak MIS-11 regional summer temperature anomaly was 2.8 °C (2.1–3.4 °C, 95% credible interval) relative to present day, with a duration of positive regional summer temperature anomalies of 16.1 kyr (14.3–17.3 kyr). The corresponding most likely peak sea-level contribution from the GrIS was 6.1 m s.l.e. (sea level equivalent) (3.9–7.0 m s.l.e.) (Fig. 2a).

The majority of the ice-sheet response lags the temperature forcing. Retreat begins around the time that temperatures above those of present day are reached in the model. When the summer temperature anomaly reaches its maximum at around 411 kyr BP, the GrIS has already lost between 1–3 m s.l.e. of ice. This is also when peak rates of mass loss of around 0.8 m s.l.e. per kyr are seen (Fig. 1). The maximum total GrIS mass loss is reached about 5–8 kyr after the peak in temperature anomalies, due to a return to temperatures colder than the present day. The simulated GrIS never reached equilibrium during the MIS-11 interglacial period, which implies that the ice sheet would have disappeared completely had the warm climate persisted. In addition, a much longer time is needed to regrow the ice sheet than was needed to melt it, even though the applied climatic forcing is essentially symmetrical. This can be attributed to the fact that the accumulation rate during the colder glacial period is much lower than the rate of melting during the warm interglacial period. In general, the lag and asymmetrical evolution of the ice sheet with respect to the forcing highlight the fact that the GrIS is a system with strong inertia and inherent hysteresis in the temperature phase space^8^,^12^.

### Comparison with proxies

The simulated magnitude of warming during MIS-11 compares well with proxy data for sea surface temperatures just off of the Southern Coast of Greenland. De Vernal and Hillaire-Marcel^4^ present a reconstruction that shows a maximum of 3.2±1.1 °C summer warming. Our simulations support the notion of such moderate warming during this time, since warming higher than 3.4 °C leads to a violation of the ice-covered GRIP constraint given that temperatures stay above present day for ca. 16 kyr ago.

The valid simulations show that southern Greenland was ice free for several kiloyears. For example, DYE-3 was most likely ice free for around 10 kyr ago between 410 and 401 kyr BP. Of a total of 0.6 × 10^6^ km^2^ land area south of 69 °N (demarcating the southern bedrock terranes), a minimum of 19% (8–37%) ice-covered area was reached, with only the high elevation mountainous regions remaining ice covered. Such a significant deglaciation in the South is consistent with the cessation of proglacial sediments for several kiloyears during this time period^5^. We also find that the minimum southern ice-covered area is essentially linearly correlated with the maximum contribution to sea level (Fig. 2b). Given the high likelihood of most or all southern ice disappearing during MIS-11 (ref. 5), this supports our upper estimate of the sea-level contribution of 6.1 m s.l.e. as being most likely. Note that this estimate remains the same even without additional weighting of simulations with more southern ice loss (see Methods), although the uncertainty increases (Fig. 2a).

A greatly reduced central dome of ice remains intact throughout MIS-11 for the valid simulations. This is consistent with the constraint of an ice-covered GRIP location^6^. In addition, our simulations show that the GRIP location stays frozen to the bed throughout the interglacial period. The contemporaneous summit migrates towards the East as the ice sheet retreats, which results in moderate ice motion in the region of the GRIP location due to deformation. This favours a slight warming in the ice basal layer. However, the reduced ice thickness also allows colder temperatures from the surface to penetrate to the base. The latter effect dominates in our simulations, resulting in temperatures in the basal ice at the GRIP location that remain below the pressure-melting point. Our simulations thus support the suggestion of Bierman *et al*.^6^ that the ice sheet remained frozen to the bedrock, given the well-preserved glacial landscape there.

### Boreal forest growth

**Figure 3:**
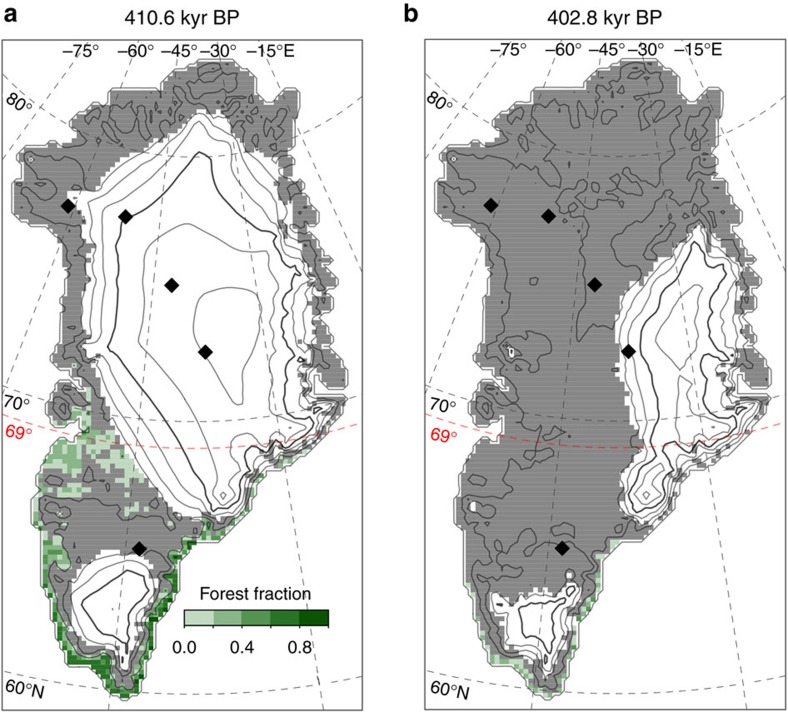
Transient change. Simulated Greenland ice sheet configuration for the most likely simulation at the time of peak regional summer warming (**a**) and the minimum ice-sheet volume (**b**). Green shading indicates the estimated boreal forest cover for each case. Black diamonds show the following ice core locations from North to South: Camp Century, NEEM, NGRIP, GRIP and DYE-3. The 69 °N parallel is highlighted (red dashed line) as the boundary demarcating the southern bedrock terranes.

The modelled climatic conditions during MIS-11 are also consistent with the growth of boreal forest in southern Greenland, particularly at the time of the maximum summer temperature anomaly (Fig. 3). Most simulations show significant forest cover along the low elevation southern coast by around 411 kyr BP, which could be the source of the pollen increase recorded in ocean sediments^4^, as well as inland on the western coast at latitudes between 65–70 °N where the ice has already retreated. DYE-3 is also ice free at this time, which would allow deposition of boreal forest DNA from nearby sources. When the ice sheet reaches its minimum extent (ca. 403 kyr BP), much less forest cover is simulated, since air temperatures over ice-free land have already started to decline by this time due to lower summer insolation.

Our simulations thus indicate that most boreal forest growth would have occurred at the peak of regional summer insolation (and summer temperatures) at around 411 kyr BP. This result is so far inconsistent with the timing of the peak pollen deposition about 5–15 kyr later in the ocean sediment record^4^. However, the dating of the core during this period could likely be improved. The age model^4^ was determined by correlating the core’s *δ*^18^O record with the global *δ*^18^O stack of Lisiecki and Raymo^13^. Comparison of the two records reveals a clear lag of the ocean sediment record of about 10–15 kyr ago during MIS-11. Correcting for this lag would bring the timing of the pollen record into close agreement with our results.

### Comparison with MIS-5e

**Figure 4:**
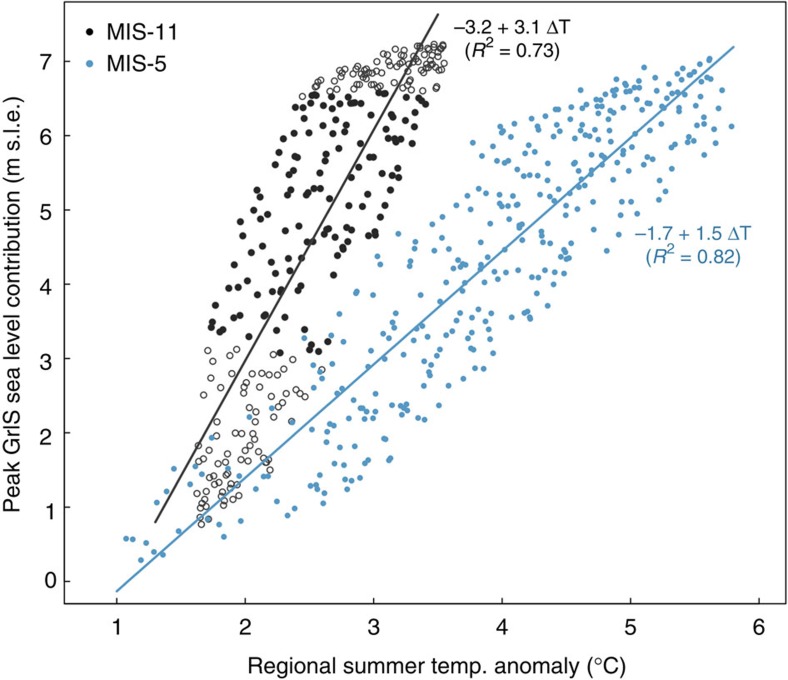
MIS-11 versus MIS-5e peak estimates. Comparison of ensembles of simulations for the MIS-11 (black points) and MIS-5e (blue points) maximum Greenland ice sheet (GrIS) contribution to sea level versus the maximum regional summer temperature anomaly. The regression lines highlight that for the same temperature anomaly, the GrIS generally loses more mass in MIS-11.

**Figure 5:**
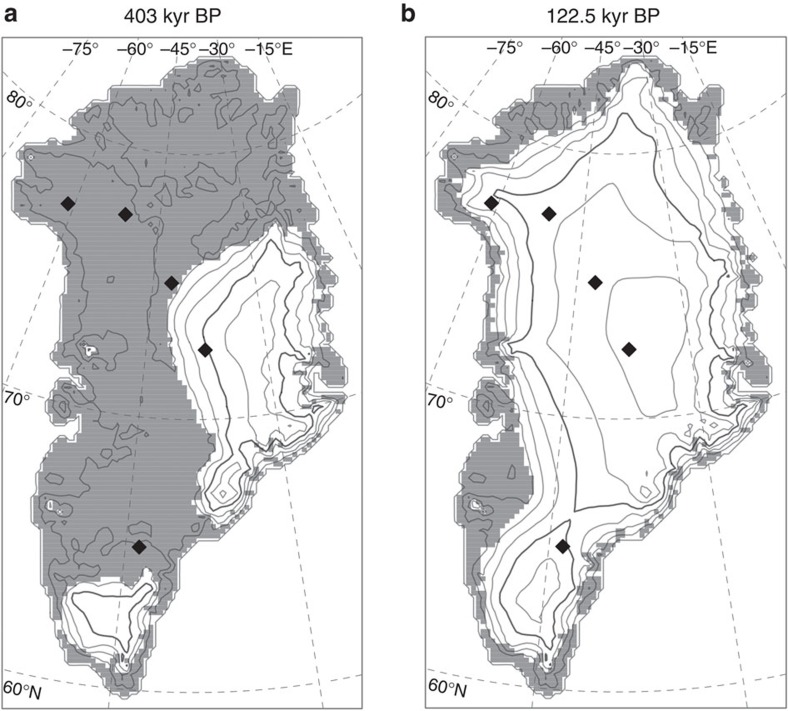
MIS-11 and MIS-5e ice sheet distributions. Minimum ice sheet distribution for MIS-11 (**a**) and MIS-5e (**b**) given the same model parameters (melt parameter *c*=−55 W m^−2^, precipitation sensitivity d*P*/d*T*=7.1% °C^−1^), and the same climatic scaling factor applied to the temperature anomalies used to force the regional climate–ice sheet model. Black diamonds show the following ice core locations from North to South: Camp Century, NEEM, NGRIP, GRIP and DYE-3. The peak simulated summer temperature anomaly during MIS-11 and MIS-5 in this simulation was 2.8 and 2.1 °C, respectively.

Our best estimate of MIS-11 regional summer warming of 2.8 °C is lower than recent estimates for MIS-5e (refs 14, 15, 16) of 3–5 °C, yet the expected mass loss of 6.1 m exceeds recent estimates for MIS-5e of between 0.6–4.3 m (refs 17, 18). More recently and using the same model as presented here, Yau *et al*.^19^ show that for MIS-5e warming of almost 5 °C, the GrIS can be expected to lose around 5 m sle. While uncertainty persists for MIS-5e, the range of all estimates is much lower than we predict for MIS-11. In fact, a comparison with analogous results for MIS-5e (Figs 4 and 5) shows that for any possible temperature anomaly, the ice sheet loses more mass during MIS-11. Given that the peak insolation anomaly during MIS-11 was lower than during MIS-5e, a larger reduction in volume in MIS-11 seems surprising.

This difference can be attributed to the unusually long duration of positive temperature anomalies during MIS-11, which allowed the ice sheet enough time to react more significantly to the climatic forcing. As hypothesized by Reyes *et al*.^5^, the GrIS becomes unstable during MIS-11 after crossing a threshold in temperature leading to melting. However, several kiloyears are needed to melt most of the ice sheet, due to the fact that only moderate levels of warming were reached. Regional summer temperatures were warmer than present day for 16 kyr in the most likely of our simulations, while for MIS-5e temperatures were warmer than present day for only around 7 kyr (refs 15, 19). The GrIS reacts immediately to warmer climates through increased melt, but it exhibits strong inertia due to its large size. Although the GrIS loses mass at rates up to 0.8 m s.l.e. per kyr, the average rate is actually closer to 0.4 m s.l.e. per kyr. Thus for the estimated level of MIS-11 warming, the ice sheet would need around 16 kyr to melt away, as is seen in our simulations.

## Discussion

These simulations are the first to quantify the GrIS contribution to MIS-11 sea-level changes from a coupled climate–ice sheet perspective, and factors not accounted for here could affect our estimates. First, the duration of the simulated interglacial period is highly uncertain, and here is perturbed around that obtained from a global climate simulation. The duration of the interglacial period around Greenland is controlled by insolation, CO_2_ and the timing of the Northern Hemisphere deglaciation. The latter, in particular, is poorly constrained for MIS-11, though it is unlikely that the interglacial period would be shorter than that presented here given other information from *δ*^18^O (refs 13, 20) and sea-level records^21^. It is clear that our temperature anomaly forcing closely follows the timing of local summer insolation anomalies over the ice sheet (Fig. 1), which could explain a return to glacial temperatures earlier than seen in Antarctica^22^. Given the inherent trade-off between warming and interglacial duration with respect to ice-sheet melting, if the interglacial period were longer in reality, this would imply a lower range of plausible temperature anomalies to achieve the same amount of ice mass loss. It is clear, however, that the temperature anomaly must have at least surpassed the threshold for stability of the ice sheet to be able to induce its long-term decline.

It should also be noted that the simulated stability of the ice sheet critically depends on changes in the modelled surface mass balance over such a long period. We have performed a large ensemble of simulations to account for model parameteric uncertainty. However, better understanding of how precipitation patterns might change during MIS-11 as a result of large-scale atmospheric circulation changes would be valuable.

Knowledge of an ice-free southern Greenland and ice-covered GRIP location constrains the plausible maximum sea-level contribution of the GrIS during this time to a range of high values approaching full deglaciation. Furthermore, the timing of peak warming at such high latitudes likely coincides with the maximum insolation anomaly in summer, peak ice mass loss rates and boreal forest growth. Meanwhile, the inertia of the ice sheet implies at least a several kiloyear delay in the maximum sea-level contribution thereafter. These results therefore provide new information as to the potential level of warming, contribution of the GrIS to sea level and its timing. In the future, palaeo evidence further corroborating the timing of any of these characteristics would go a long way to further reducing uncertainty in our understanding of the coupled climate–ice-sheet system.

## Methods

### Model

We use the coupled climate–ice sheet model REMBO-SICOPOLIS^9^,^10^. SICOPOLIS is a 3D polythermal shallow-ice approximation ice-sheet model^23^ run at 20 km resolution. REMBO is a regional energy-moisture balance model that simulates the climate and surface mass balance over Greenland at the same resolution, with prescribed temperatures over the ocean. REMBO uses ECMWF reanalysis data^24^ as boundary forcing for the present-day climate, to which spatially-constant monthly temperature anomalies are added in transient palaeo simulations. Time series of sea-level changes and atmospheric CO_2_ concentrations are also prescribed to account for marginal forcing of the ice sheet and changes in the radiative forcing of CO_2_, respectively. Insolation changes are calculated internally by the model using transient orbital parameters^25^. The time series of monthly temperature anomalies, sea level and equivalent CO_2_ were obtained from a transient global simulation of the last 8 glacial cycles^26^.

The fraction of land covered by boreal forest was calculated offline using the vegetation model VECODE^27^,^28^. Here, VECODE was used to calculate the expected equilibrium vegetation coverage for a given time slice, given the annual mean temperature, annual mean precipitation and growing degree days (GDDs) as input.

### Uncertainty

The set of simulations performed here comprise a perturbed physics ensemble. We varied two key model parameters that affect the surface mass balance of the ice sheet (melt and precipitation scaling parameters), consistent with the ensemble presented by Robinson *et al*.^8^ and Yau *et al*.^19^. The ensemble of model versions exhibits a broad ice-sheet sensitivity to climate change. The melt parameter *c* appears in the insolation-temperature melt equation^9^,


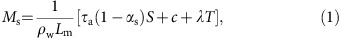


where *M*_s_ is the melt rate, *ρ*_w_ and *L*_m_ are the density of water and the latent heat of melting, respectively, *τ*_a_ is atmospheric transmissivity, *α*_s_ is surface albedo, *S* is insolation at the top of the atmosphere and *c*+*λT* is a linear parameterization of the long-wave radiation and turbulent and latent heat fluxes as a function of near-surface temperature *T*. The parameter *c* was assigned a Gaussian prior of −55±2 W m^−2^, while the modelled sensitivity of precipitation to temperature change was assigned a prior of d*P*/d*T*=6.3±3.3% °C^−1^ and scaled internally in the REMBO climate model. The priors were determined through comparison with regional and global GCM simulations under present-day and global warming scenarios^8^,^10^.

As mentioned above, the regional climate model is driven by a time series of monthly temperature anomalies over the surrounding ocean calculated in a global transient glacial-interglacial simulation^26^. We consider the long-term time series of temperature anomalies as a robust forcing signal, as it is strongly driven by changes in orbital forcing (Fig. 1). However, very little data exist to constrain the duration and magnitude of warming during MIS-11 around Greenland. For this reason, we also applied a free scaling parameter to adjust the boundary conditions during the interglacial period, while maintaining the shape and timing of the original curve. This allowed us to force the ice sheet model with time series exhibiting a wide range of plausible interglacial temperature anomalies, and to better quantify the uncertainty of our approach.

We thus accounted for this climatic uncertainty in the simulations by incorporating a scaling factor applied to temperature anomalies during the interglacial period. The scaling factor, *f*_s_, was used to modify the original temperature anomaly time series proportionally to its maximum value, d*T*_orig,max_, such that


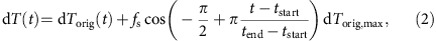


where *t*_start_ and *t*_end_ are the times of the beginning and the end of the interglacial time window. In this case we applied this scaling to the period with summer temperature anomalies above −2.0 °C in the original time series, corresponding to times between −424 kyr BP and −395 kyr BP. The width of this window and the cosine scaling was chosen to avoid artifacts in the time series related to the application of the scaling perturbation only during the period of positive temperature anomalies (that is, the method ensures a smooth transition between the original time series and the perturbed section of the time series). The scaling factor *f*_s_ was given an even prior (all values equally likely). The parameter value combinations used in the simulations were then obtained using Latin Hypercube sampling with a total ensemble size of 300 members.

The ensemble of simulations was constrained by first eliminating any simulation from consideration that did not satisfy the hard constraints of an ice-covered GRIP location at all times and an ice-free DYE-3 location at some point during the simulation. Based on palaeo data^5^, we further assume a likelihood weighting function that gives higher weight to simulations with less ice cover in southern Greenland. The likelihood weighting function used in our ensemble was


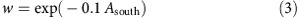


where *w* is the non-normalized weight of the simulation and *A*_south_ is the ice-covered land area below 69 °N (%). The weight is 1 when *A*_south_=0 and it descends towards zero as the ice coverage increases. The exponential coefficient of 0.1 was chosen such that the weight approaches zero at around 50% southern Greenland ice coverage, which is consistent with the ice-free DYE-3 constraint. The weighting function is shown in the inset of Fig. 2b. To ensure that our overall conclusions are robust, we tested various coefficients, as well as no additional weighting (also shown in Fig. 2b), with similar results.

The probabilistic information presented in this paper was calculated from a weighted Kernel density estimate (KDE) of the probability distribution, given the prior and posterior weighting as described above. We used the R package ‘ks’^29^ to calculate the 2D KDE for the variables maximum summer temperature anomaly and maximum sea-level contribution. The 2D density estimate was used to determine the 95% credible intervals and the most likely simulation.

### Data availability

All numerical code used in this study, analysis scripts and the model results are available from the authors upon request.

## Additional information

**How to cite this article:** Robinson, A. *et al*. MIS-11 duration key to disappearance of the Greenland ice sheet. *Nat. Commun.* **8,** 16008 doi: 10.1038/ncomms16008 (2017).

**Publisher’s note**: Springer Nature remains neutral with regard to jurisdictional claims in published maps and institutional affiliations.
